# Lessons learned from academic medical centers’ response to the COVID-19 pandemic in partnership with the Navajo Nation

**DOI:** 10.1371/journal.pone.0265945

**Published:** 2022-04-05

**Authors:** Tasce Bongiovanni, Sriram Shamasunder, William Brown, Cristina Rivera Carpenter, Matthew Pantell, Bassem Ghali, James D. Harrison

**Affiliations:** 1 Department of Surgery, University of California San Francisco School of Medicine, San Francisco, CA, United States of America; 2 San Francisco General Hospital, Trauma and Acute Care Surgery, San Francisco, CA, United States of America; 3 Division of Hospital Medicine, University of California San Francisco School of Medicine, San Francisco, CA, United States of America; 4 HEAL Initiative, Health, Equity, Action & Leadership, San Francisco, CA, United States of America; 5 Department of Epidemiology and Biostatistics, University of California San Francisco School of Medicine, San Francisco, CA, United States of America; 6 Department of Medicine, Division of Prevention Science, University of California San Francisco School of Medicine, Center for AIDS Prevention Studies, University of California, San Francisco, CA, United States of America; 7 Bakar Computational Health Science Institute, University of California, San Francisco, CA, United States of America; 8 Department of Nursing, Northern Arizona University, San Francisco, AZ, United States of America; 9 Department of Pediatrics, University of California San Francisco School of Medicine, San Francisco, CA, United States of America; The University of Tennessee Knoxville, UNITED STATES

## Abstract

**Introduction:**

Structural forces that drive health inequalities are magnified in crises. This was especially true during the COVID-19 pandemic, and minority communities were particularly affected. The University of California San Francisco and Health, Equity, Action, Leadership Initiative jointly sent volunteer teams of nurses and doctors to work in the Navajo Nation during the COVID-19 pandemic. This presented an opportunity to explore how academic medical centers (AMCs) could effectively partner with vulnerable communities to provide support during healthcare crises. Therefore, the aims of this study were to describe volunteers’ perspectives of academic-community partnerships by exploring their personal, professional and societal insights and lessons learned based on their time in the Navajo Nation during COVID-19.

**Methods:**

We recruited key informants using purposeful sampling of physicians and nurses who volunteered to go to the Navajo Nation during the spring 2020 COVID-19 surge, as well as hospital administrators and leaders involved in organizing the COVID-19 efforts. We used in-depth qualitative interviews to explore key informants’ experiences pre-departure, during their stay, and after their return, as well as perspectives of the partnership between an AMC and the Navajo Nation. We used thematic analysis to systematically identify, analyze and report patterns (themes) within the data.

**Results:**

In total, 37 clinicians and hospital administrators were interviewed including 14 physicians, 16 nurses, and 7 health system leaders. Overall, we found 4 main themes each with several subthemes that defined the partnership between the AMC and the Navajo Nation. Mission and values incorporated civic duty, community engagement, leadership commitment and employee dedication. Solidarity, trust and humility encompassed pre-existing trust, workforce sustainability, humility and erasure of ‘savior narratives.’ Coordination included logistical coordination, flexibility, selectivity of who and what traveled to the response and coordination around media response. Workforce preparation and support encompassed understanding of historical context and providing healthcare in limited settings, dangers of inadequate preparation and the need for emotional support.

**Conclusion:**

This study provides guidelines which AMCs might use to develop and improve partnerships they have or would like to develop with vulnerable communities. These guidelines may even be broadly applied to partnerships outside of a pandemic response. Importantly, such partnerships need to be built with trust and with an eye towards sustainability and long-term relationships as opposed to ‘medical missions’.

## Introduction

The novel coronavirus (COVID-19) pandemic laid bare the large, systemic, and pre-existing racial inequities in the United States, [1] and exacerbated health inequalities in many communities. Native American communities were hit especially hard by the COVID-19 pandemic. Death rates in the Navajo Nation were 10 times higher than in the neighboring state of Arizona, and throughout most of the pandemic, these rates were higher per capita than any state [2–4]. These numbers may be underreported because multiple state databases must be accessed to determine the incidence of COVID-19 given the expanse and location of the Navajo Nation [5]. In a report from the winter of 2021, Navajo comprised 26% of the population in Coconino county where the Arizona portion of the Navajo reservation is located, but suffered 77% of that county’s Covid-19 deaths [6]. Although rates varied depending on which COVID-19 surge was occurring, American Indian, Alaska Native and Native Hawaiian persons had the highest incident cases and deaths per 100,000 population of any race/ethnicity in the United States [7]. Throughout the country, Native Americans were more likely to be hospitalized and die than White patients [7]. This remained true over every ‘surge’ or sharp increase in cases. The toll of the pandemic was compounded by centuries of colonialism leading to a lack of funding and limited access to healthcare, in addition to geographic isolation, lack of infrastructure and already deprived living conditions. Additionally, a strength of the Navajo Nation, living in multigenerational homes, helped the virus spread quickly when many families were infected within the same household. Many of these systemic issues were already threatening the Navajo, or Diné, culture and livelihood.

The COVID-19 pandemic accelerated the need for healthcare systems to act to address these health inequities. Specifically, academic medical centers (AMCs) are large conglomerates of resources and clinicians with the ability to affect change in social determinants of health. While AMCs serve important roles in the healthcare system in the United States, such as encouraging innovation, research, and by educating and training the next generation of clinicians, [8] they have begun to face criticism for a lack of focus on health inequalities within local communities and at a population level [9]. Furthermore, AMC-related community work has been criticized as too focused on what AMCs need, research for example, and not on what the community needs, resulting in renewed interest in community engagement and integration [10]. One potential barrier to AMC-focused work with vulnerable communities is a lack of guidance about how to operationalize such a partnership. While there are descriptions of nonprofit physician groups undertaking work globally, focused on ‘medical missions,’[11–13] an understanding of how to complete this type of work with populations geographically located within the United States is lacking.

In response to Navajo Nation President Nez’s call for health worker reinforcements due to the COVID-19 surge in April 2020, [2] the University of California San Francisco (UCSF) and Health, Equity, Action, Leadership (HEAL) Initiative jointly sent volunteer teams of nurses and doctors to serve in three Navajo Nation hospitals, starting with volunteers who first went as a group in April 2020, and groups continued to be present through spring of 2021. This presented an opportunity to understand how AMCs could effectively partner with vulnerable communities to provide support during times of healthcare crisis. Therefore, the aims of this study were to describe volunteers’ perspectives of academic-community partnerships by exploring their personal, professional and societal insights and lessons learned based on their time in the Navajo Nation during COVID-19. These findings can be used to inform future partnership development between AMCs and vulnerable communities.

## Methods

### Definitions

Health inequality and health inequity both are critical threads throughout our study. Whereas health inequality refers to the uneven distribution of health in or between populations, a situation that is sometimes unavoidable, health inequities “may be thought of as the presence of systematic disparities in health (or its social determinants) between more and less advantaged social groups”[14].

### Setting

The setting for this study was UCSF, a large quaternary academic medical center. Within UCSF, the HEAL Initiative aims to train, transform, and build a community of front-line health workers committed to serving resource-denied people who are medically underserved locally and internationally. HEAL seeks to contribute to the movement for global health equity led by communities themselves [15]. Since 2015, HEAL has supported both site fellows (those already embedded in the community employed at the worksite) and rotating fellows (US-based physicians and nurses) to learn and work together at four different sites within the Navajo Nation over two-year fellowship time periods. Nineteen of these 53 non-Navajo fellows have stayed in the Navajo Nation to work after their fellowship. Further, HEAL is part of a United States Government Accountability Office commission as a strategy to address workforce vacancies. The Navajo Nation is the largest reservation and sovereign nation within the United States. It geographically spans Arizona, New Mexico and Utah, with land also in Colorado, states which had varying rules and protections against COVID-19, making tracking and tracing challenging and the sovereignty and leadership of the Navajo Nation imperative [16].

The volunteers were deployed across five hospitals, working in emergency departments, inpatient wards, hospital admissions, covering swing shifts, staffing intensive care units, and checking on patients who were isolating in hotel-motel isolation programs.

### Study design and oversight

We used qualitative in-depth interviews which generate rich narrative data about individual attitudes, expectations and complex social experiences and interactions [17]. The UCSF Institutional Review Board approved this study.

### Participants

We recruited key informants using purposeful sampling methods [18]. We identified UCSF physicians and nurses who volunteered to go to the Navajo Nation during the COVID-19 surge starting April 2020, as well as hospital administrators and leaders involved in organizing the COVID-19 effort. Participants were invited by email and given general information about the study. We interviewed participants until achieving “theoretical saturation,” the point at which no new concepts emerge from reviewing successive data from a sample [18]. We did not interview patients or clinicians based at the Navajo Nation because of the time sensitivity of this work and the IRB regulations of the Navajo Nation.

### Data collection

We developed a de novo interview guide informed by previous studies of medical mission trips, global health partnerships and clinician experiences from prior pandemics [11, 12, 19–22]. Interview questions focused on participants’ understanding and experiences pre-departure, during their stay, after their return, and perspectives of the partnership between an AMC and the Navajo Nation (S1 File). Questions were open-ended, allowing participants to direct the course of discussion and probes were used to clarify concepts and elicit detail [18]. Interviews took place over the online platform Zoom and were led by two members of the research team (TB, JH) who are trained in qualitative interviewing techniques. Each interview was digitally recorded. All participants provided verbal consent at the start of each interview.

### Analysis

Interviews were professionally transcribed and verified for accuracy. We used thematic analysis to systematically identify, analyze and report patterns (themes) within the data [23]. At least two members of the research team independently performed open coding of transcripts using a data driven (inductive) approach. To ensure methodological rigor, the research team met at regular intervals during analysis to develop a code book and to resolve any coding discrepancies using negotiated consensus [24]. Codes were then grouped into higher order themes. We used Dedoose Version 8.0.35 (Los Angeles, CA: SocioCultural Research Consultants, LLC) for data management.

## Results

In total, 37 key informants were interviewed: 14 physicians, 16 nurses, and 7 health system leaders. This cohort included 23 women and 14 men. The average time for the interview was 60 minutes with a range of 30–75 minutes.

**Fig 1:**
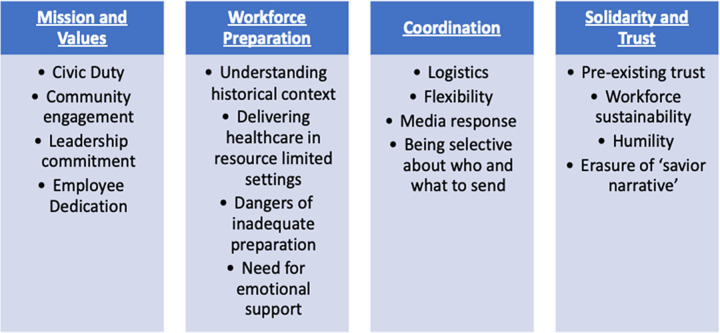
Overview of themes.

Four themes characterized the partnership between an AMC and the Navajo Nation: (1) Mission and values, (2) Solidarity, trust, and humility, (3) Coordination and (4) Workforce preparation and support (Fig 1). Each theme had supporting subthemes (Table 1, Fig 1). Representative quotes for each follow below. Other contributing quotes have been included in a supplementary file (S2 File).

**Table 1:** Themes, subthemes and recommendations.

Theme	Subtheme	Recommendations
Mission and values	Civic duty	• AMCs have a civic duty to partner with diverse and vulnerable communities • Recognize importance of AMCs in public health crisis
	Community engagement	• Engage in partnerships because it’s a core value of the institution (not for publicity)
	Leadership commitment	• Active leadership engagement for planning and for volunteer support • Leadership commitment to the values of the institution, including community engagement
	Employee dedication	• Successful partnerships support the values and priorities of employees and makes employees proud of the institution
Solidarity, trust and humility	Pre-existing trust	• Build deep partnerships now (HEAL being an example), before crisis, so that if assistance is needed you have trust, respect and understanding to build on • Trust is built by prior commitments which have been fulfilled
	Workforce sustainability	• Ensure partnership and commitment continues after the crisis–individuals may change but the institutional presence continues • Commit staff/trainees/faculty over a longer time frame to magnify the impact during and after crisis • Fund such volunteer efforts with FTE so that staff are not using vacation time • For those interested in social medicine, allow it to be part of career advancement
	Humility	• Listen to partners–collaborate on their needs and what you have to offer • Listen to and learn from the community, center their voices • Do not replicate colonial dynamic of highly powered institutions dictating agenda; flatten power hierarchies
	Erasure of ‘savior narratives’	• Solidarity is key • Understand the savior narrative in order to avoid it • Align with the mission of showing up and support the resilience of the community in managing the crisis to date • Thank the community for hosting
Coordination	Logistical coordination	• There will always be interest. The challenge is not finding people but organizing them • Logistics is key and a team and process are needed to streamline paperwork, housing and transportation. • Small issues can be big problems in the context of different languages, lack of internet/phone service and trying to solve issues across time zones • Important HR workflows and policies include paperwork (and burden on local partners), ensuring coverage for people leaving from AMC, re-entry process workflows (labor and employee relations for 14-day reentry support/quarantine, and the applicable policies) • Temporary assignment protocol by leadership for payment (or nonpayment) • Consider multiple stakeholders: occupational health, PPE suppliers, Occupational Health, Spiritual Health, Academic Affairs, Credentialing, professional services agreements, Worker’s Compensation, and professional liability
	Flexibility	• Partners during crisis are under tremendous pressure already; volunteers should be ready to contribute in whatever way is helpful • Limit expectations • Volunteers should be willing to work in different departments than the one they currently work in • Be ready to work hard • People who cannot adapt to working outside of area of expertise and comfort (but still within scope of practice) may need to be screened out
	Selectivity of who and what traveled to the response	• Avoid impulse to send more ‘stuff,’ as it becomes challenging to manage, store, track and dispose of when not use • People screened need to be flexible–flexible in shift, role and tasks and open-minded in expectations, based on needs of local partners. • People who volunteer need to be committed to protect the relationship
	Coordination around media response	• Beware of the media narrative–can slip into savior narratives and often overlooks the efforts and resilience of the host community • May highlight social determinants, but does not expose the structural oppression • Beware of focus on the visiting providers, rather than the people who have for years lived and worked in the communities under difficult circumstances and pressures
Workforce preparation and support	Understanding historical context	• Historical context of current health disparities • History of the people, the social differences and why they exist, the structural analysis of the health disparities, the healthcare system and how it compares/contrasts to the one we work in • Anti-racism, anticolonialism–unbuilding systems of structural violence
	Understanding healthcare in resource-limited settings	• Understanding social determinants of health • Integrating partners with local expertise and context
	Dangers of inadequate preparation	• Pre-departure preparation is nice–note that operationalization in such situations occurs quickly (hours-days) • Training in ethical dilemmas and challenging scenarios in resource-constrained settings was desired • Without detail about the particular patient population there is a risk for approaching patients insensitively • Concern that without knowledge of past wrongs, those same harms may be perpetuated
	Need for emotional support	• Recognize emotional burden on individuals when working in crisis in unfamiliar space • Importance/value of support from the AMC, including the ability to immediately access coordinators • Weekly (or more often) debrief calls to allow space for volunteers to process, bring in community member who can provide local context and answer question • Weekly check ins attended by leadership • Social isolation challenging in context of social distancing

(1) **Mission and values****: Alignment between the mission and values of the AMC with the crisis response effort is essential and has great benefits at both the organizational and individual levels.**

### Civic duty

AMCs are thought to have a civic duty to health equity, to create and sustain partnerships with diverse and vulnerable communities.

Most people see AMCs as highly technical, complicated places that you want to have around if you have a very complicated disease that requires huge expertise and maximal technical experience. They don’t really appreciate… how important they are in a public health crisis. To be a hub-and-spokes-type relationship with the academic medical centers being a hub but having spokes out into the community and particularly to the rural community where hospitals are.

Come with an open mind but also come to engage in these partnerships, not to advertise them and not because the historical moment demands them but because it’s part of the culture and values of the institution or the system.

### Community engagement is a core value

AMCs must be willing to commit to community engagement as a core value, for the value’s sake, and not for positive publicity.

The academics who want to get involved, get involved. Not just during when it’s cool to do it… but, you know, chairs hopefully will get engaged when there’s not just the potential for a—a good tweet or a—a spot on the local news.

### AMC leadership must be committed to activities that fulfill the mission and values of the organization

They must be actively involved in, and show commitment to, the values espoused by their organization related to community engagement and health equity.

We did a great job preparing [for the pandemic], but we then were able to kind of shift the focus to be external, like "What else is going on out there? Who else might need help?" That is rare… I think it’s something you can encourage in other institutions, but it is built in… to the people, to the leadership. I think that it speaks volumes that, instead of hoarding and protecting what is ours, we were able to give freely and encourage service elsewhere.

### Employees want to live out these values

Employees’ individual values act to reinforce both the partnership and the organizational values, while also strengthening internal bonds between employees and their institution.

Going in to COVID I wasn’t sure that UCSF was aligned with my values. I wasn’t sure this was my forever academic home. And after seeing UCSF’s response to Navajo Nation, or after being a provider there myself and feeling so supported by UCSF in undertaking this work, this has fundamentally changed how I feel about UCSF as an organization and as a community that I am so proud to be a part of. For me, and I hope for our UCSF leaders, that is worth the investment. That ability to rekindle the sense of community at UCSF and that ability to demonstrate that we can put our values that we have on paper into action.

(2) **Solidarity, trust and humility****: Participants felt that building partnerships based on solidarity and trust was a cornerstone to the crisis response effort. Participants also advocated for a sustainable commitment to the Navajo people beyond the crisis. For success, partnerships require humility of the AMC and erasing the ‘savior’ narrative.**

### Pre-existing trust from Navajo Nation partners

The rapid pandemic response was possible due to the pre-existing relationship with the Navajo Nation. This commitment was trust-building.

For organizations who want to leverage their own power to be there in solidarity with partners in resource-denied settings—the lesson is, build those deep partnerships that are real now. So that when your assistance is needed down the road, you’ll be able to not try to build five years of trust in a day. But they’ll be able to respond in the way that the partner would respect and would feel that it’s value in that moment.

### Creating workforce sustainability within engagement plans

Participants advocated that the partnership would be sustained, achieved by longitudinal workforce planning that went beyond relying on volunteerism.

I think the key is continuing the partnership. It needs to be support that is provided not just during this…worldwide crisis, but somehow ongoing. I think everyone wants to help right now and, you know, the house is burning down, everyone’s going to be there trying to help, but there are issues that are happening even though the world isn’t paying attention, and I think you must be there for those moments, as well to show that you truly are invested and truly are walking in solidarity.

Rather than looking to fit in holiday time or vacation time but to really honor it and create a job that is funded adequately and that is providing benefits so that it’s not a tremendous ask for people to go…and it’s part of your FTE. I would say, sign me up. Where do I sign? I would happily do that. So creating it as part of my job, my career, making it part of my advancement—

### Humility is key

To achieve solidarity, participants had to first understand and embrace humility. Power hierarchies needed to be flattened, through humility on the part of all clinicians and leaders from the AMC.

I think a lot it is just focused on listening to your partners… I think so often, and so much more common than our model at HEAL, is what really ends up being like a replication of a colonial dynamic of very highly powered institutions—academia—with a lot of expertise in some frame, going in and saying, “Hey we have these people; they’re highly skilled. We’re going to send them to you, and we want them to do X, Y, and Z…” And, you know, that looks like the highly powered institution calling so many of the shots. Which is really, in essence, a repetition of how global health has been done wrong, in many areas over history.

Remind people to be humble. Humility, I think, is something that—because we’re experts, we sometimes forget how to be humble. And we barge in, then we replicate the cycle that’s been done in the past. But I think if you are humble and open to what they have to offer and see how that can be incorporated into what we have. It’s bridging the two, together.

### “Savior narratives” need to be erased

AMCs need to avoid a savior narrative (a common trope wherein a white protagonist is portrayed as a messianic figure who rescues non-whites from unfortunate circumstances) [25].

It was really helpful to have a lot of background work from HEAL, and to not have the whole "white savior" narrative going on, and not have that whole dynamic of: "Well, we’re going to come in. We are from this great medical center with all our great knowledge, and we’re going to impart all this knowledge on you." It was more like: "We have more extra hands. We’re here to help you right now for a short period. We’ll do what we can. We’ll do what you want us to do." I just made a point when I first got there—and I kept trying to remind myself to do it, too: When people would thank me for coming, I would say: "Thank you for letting us be here." And I really meant it.

(3) **Coordination****: Many respondents spoke about the role of AMCs in making administrative, workforce and logistical arrangements related to travel, lodging, supplies and credentialing of clinicians. This included coordinating human resources issues and support of clinician time away from their primary site of work.**

### Coordination of the crisis response

High quality administrative and logistical support is essential for rapid deployment of clinicians.

The challenge will never be interest. It will never be a lack of people who are willing and highly interested in doing this work. The logistics are always key. Originally, I was going to be able to go for a week, and that seemed like it was going to be enough, but then they’re like, "No, no. You have to come for at least two weeks or else it’s not worth it to go through all the paperwork."

I think one of the biggest things is getting the logistics right, and really thinking through all of those human resources workflows. Because small things become big things when you’re in another community that speaks another language, and you don’t have good internet, and you don’t have good phone service. Those small things can become big things that can affect your ability to be able to focus on your job and focus on the reason why you’re there.

### Flexibility

The workforce needs to be willing to work wherever, and however, they are needed by the community health system.

Folks … were passed into other departments—their high degree of training and expertise and their flexibility and resilience were able to be slotted into different departments than the ones that they are in at UCSF. And so, A) go wherever you’re needed, and then B), once you’re there, do whatever is needed. It isn’t time to question protocols, it isn’t time to mention the way this would be done at UCSF, it’s the way to be alongside and support.

### Selectivity about who and what is sent

AMCs need to make sure they are sending the appropriate personnel and equipment needed by the partner.

I could tell from the people who told me, “Well, I want this certain shift and I want this certain [thing].” “No, you can’t go then because you think it’s going to be like here, and that’s not going to be the case.” For the Navajo Nation, I would just reject people [during screening] because I just said, “You’re not ready for this.” Because you must do what is needed at the end of the day.

[It’s possible that] what’s most useful is to help organize the PPE closet and make sure everything is clean and easily accessible. And to do that, you know, 10 hours a day. And for some people that’s a non-starter.

### Media preparation

Clinicians struggled with how to respond to the media. AMCs need to help coordinate and prepare clinicians for potential media enquiries so that the mission and narrative are not usurped by the media.

Watching the news clip, they literally took what I said out of context to fit the narrative of how they wanted to portray what was happening in the Navajo Nation. I thought that was really concerning because it wasn’t portraying an accurate picture of the situation; it was more in terms of, “Look at how these people from San Francisco are coming in to save the day.” … [This] really devalues the resilience and the integrity of the Navajo Nation and what they’ve experienced during this pandemic.

Most of it was pretty cringey. When we got to the airport in Albuquerque there was a reporter who interviewed a couple people. They created this story: “Oh, these great, awesome medical people are here from San Francisco.” It was the total “white savior” thing.

(4) **Workforce preparation and support**: **Pre-departure preparation of the workforce was critical. This included ensuring clinicians are educated about the historical context, culture, healthcare delivery system, and health inequalities that exist in the community. Further, preparation was needed on concepts of health equity, solidarity and the savior narrative. Support was also essential.**

### Understanding the historical context of the community

Clinicians expressed the need to understand the historical context of the Navajo Nation and its relationship with the United States.

When you’re coming into an area, you do need to understand not only current but historical context, especially with things like COVID because COVID rates here didn’t occur in a vacuum. There’s a lot of systemic violence that has happened over a very long time that has created these conditions. Nothing occurs in a vacuum, and we tend to be very ahistorical and acontextual. So, what is the historic and current context?

There was one [background reading] about cultural humility that totally shifted my perspective on where we were going and why we were going there and how to be there with people. I think giving people an explicit framework about just not going in expecting this very hierarchical typical role.

### Understanding healthcare in resource-limited settings

Clinicians desired preparation on providing healthcare in resource-limited settings.

One example is the electricity issue, so some patients have electricity that is there all the time, and other patients they live in homes where they have a generator that will give them electricity. And this is important because the oxygen compressors that we would send people home with you have to have electricity all the time, it can’t be dependent on a generator because if the generator fails then you can’t just like not be without oxygen if you’re dependent on it. Understanding some of that living situation was really important.

### Dangers of inadequate preparation

Some participants expressed that a lack of preparation could lead to harm through perpetuating past wrongs through misunderstandings of culture and context.

I wish we had known more about this patient population in more detail prior to coming because we probably said a lot of things that maybe weren’t appropriate. We probably approached patients in a way that we didn’t realize was not the most sensitive just simply because we didn’t know. I think having a very comprehensive understanding of what patient population you are serving and what their needs are and how you as a clinician can observe and modify your clinical practice to be more sensitive to the needs of that community would be really, really helpful.

### Provision of emotional support

Providing emotional support for clinicians while working is essential but difficult during a pandemic.

We quickly learned the value in the support that we were giving to our volunteers, and we really tried to hold ourselves honorable to that. I think that was probably one of the biggest learnings that we had quickly, was that it was really valuable for them to be able to just email me or text me, and for me to be able to take care of something for them. We also tried to set up regular weekly check-ins with senior leadership–the Chancellor and CEO and President participated in [these], and to make sure that we were hearing about what was happening on the ground with them so that we’d be able to respond to anything that was coming up. We really, really saw the value of just having such close contact with the volunteers.

Thinking about a pandemic and sending your providers to an unfamiliar space you must make sure that they’re going to be prepared for how isolating that may be. And figure out a way to give them a community and a safe space.

## Discussion

In this study, we investigated how AMCs could effectively partner with vulnerable communities to provide support during crises. While much work has been done on relationships outside of the United States, few studies have examined the role of AMCs in partnership with populations geographically located within the United States. Using qualitative methods, we explored volunteer perspectives of the academic-community partnership during the COVID-19 response and aimed to define personal, professional and societal insights and lessons learned. We found four overarching themes: (1) Mission and values, (2) Solidarity, trust and humility, (3) Coordination and (4) Workforce preparation and support. Each theme could be further subdivided into supporting subthemes.

Some of our findings echo those reported in other studies. For example, our finding that employee pride and engagement in their institution is reflected in studies that show field experience having a positive influence on the careers of students and increase engagement in service, though in our study this pride increases the bond with the AMC, and is not just limited to the individual [26]. The need for preparation of AMC employees through historical and contextual education in our study is reflected in a similar call for attitudinal development in international work [22]. Finally, the need to have thorough logistic planning, the need for flexibility and the need for long-term relationships, as has been reported in a well-cited framework for short term trips, [19] was also reflected in our study.

Our study was unique in that the partnership was geographically within the United States, with an AMC as opposed to a non-profit group, and not considered to be a ‘medical mission’ but an act of solidarity during a global pandemic. People needed to be willing to do what was necessary even it was outside of their specialty or expertise at UCSF in order to be effective. Humility was key, and even after careful screening and pre-departure education, there were still circumstances in which volunteers found themselves having accidentally overreached by being abrasive or less culturally appropriate than they may have wanted to be. The support and frequent meetings were crucial to address and process these events. The need for AMCs to embrace their mission and values incorporated in civic duty, community engagement, as well as leadership commitment and employee dedication to any community partnerships, also appear unique to our study. Also unique was our finding that a deep understanding of historical context of disenfranchisement and inequality of Native American communities is incredibly important. Being prepared to deal with the media, in order to appropriately shape the partnership narrative, is an important lesson that we have not seen published previously, and presented itself as an issue both internally to the AMC, on departure, and while volunteers were serving on the Navajo Nation. Many participants expressed a deep discomfort with dealing with the media at all. Finally, the COVID-19 pandemic being respiratory in nature, with strict masking and social distancing precautions, affected the social support needs volunteers reported, as well as their ability to engage with and interact with the community, often seen as the greatest benefit of these partnerships on all sides. To adjust, AMC and HEAL leadership provided regular virtual support, which was often not enough given the emotional challenges many volunteers faced.

Our study has several limitations. First, because we could only include UCSF volunteers—and not also people already on the ground working on the Navajo Nation—our findings are limited to a subset of people who are transient to the work. However, any AMC partnership will necessarily include people transient to the work and therefore this is an important subset to understand. We recommend work be done to also understand the host side of the academic-community relationship in this context. Another limitation is that people from AMCs with a different culture to UCSF, including the values and mission of the leadership, would likely report different experiences, resulting in different themes emerging. Consequently, the experiences and themes described in this study are representative of one partnership and one place, and therefore may not be generalizable to other communities or regions of the country.

Viewed with those limitations in mind, our study can offer key considerations for AMCs across the country to engage and partner with vulnerable communities, acting in solidarity to enact the mission and values of the host and partner organization (Table 1). First, as AMCs become more involved in their local communities, learning from these experiences is an important contributor to longer-lasting partnerships and improved outcomes for both partners. Second, building a sustainable and long-term plan for partnership requires listening carefully and making the conversation a two-way planning process. This partnership was based on close relationships with specific members of the community who served as key advocates and communicators with the volunteer groups. Third, volunteers should be vetted carefully and given thorough education not only about providing healthcare in under-resourced settings, but also the historical and cultural context in which they will serve, and importantly, approaching the partnership with humility and openness to learn. Five times more people were interviewed than were accepted and volunteers were screened out who did not appear to be humble or have a teamwork orientation. Fourth, logistics were a huge challenge given the number of people, sites, credentialling and onboarding that needed to occur, including testing and quarantine upon return to the home AMC, something unique to a pandemic response. These suggestions can help guide AMCs who are setting up relief responses to COVID-19 and future pandemics. The themes and subthemes we describe may even represent principles which can be broadly applied to partnerships outside of a pandemic response, such as those addressing healthcare inequalities.

## Conclusions

In conclusion, our study highlights that some employees of AMCs yearn for service and partnership, and that these partnerships should be part of the foundation of an AMC’s mission in service to its communities. We hope that deep, meaningful, longstanding partnerships with AMCs and communities deprived of their health rights continue to be developed in the spirit of the quote attributed to Aboriginal rights groups in Queensland, “If you have come here to help me, you are wasting your time. But if you have come because your liberation is bound up with mine, then let us work together” [27].

## Supporting information

S1 File Supplementary information interview guide. (DOCX)

S2 File Supporting quotes. (DOCX)
