# Supplementary material for: Lessons learned from academic medical centers’ response to the COVID-19 pandemic in partnership with the Navajo Nation
Source: PLoS One. 2022 Apr 5;17(4):e0265945. doi: 10.1371/journal.pone.0265945 (PMC8982841; doi:10.1371/journal.pone.0265945)
Supplement: S1 File — (DOCX) [file pone.0265945.s001.docx]

**S1: Supplementary Information: Interview Guide**

**Interview Guide: Volunteers who traveled**

**Introductions**

Overview of the project and aims

**The Basics**

- What did you know about the Navajo Nation before you went? Had you worked there before?
- We all have a personal/individual narrative, a story, about who we are and why we do things. What made you decide to travel to the Navajo Nation?
- What is your understanding of the causes of the COVID-19 outbreak on the Navajo Nation before you went? And after?

**Narrative & Partnership**

- The HEAL initiative talks about walking in solidarity with communities. Can you tell us about how that statement relates to your experience in the Navajo Nation?
- Do you think providers, patients and the community perceived that USCF and UCSF providers were walking in solidarity? Or did they perceive something different?
- You’ve told us why you went to the Navajo Nation. Once on the ground at the Navajo Nation, can you identify and describe an event or experience that shifted your narrative about your role there?
- Did you feel like you were partnering with other providers on the ground? With the Navajo people? What factors or experiences facilitated a successful partnership? What made it unsuccessful?
- What mistakes did you make? What could have gone better?
- What about the media/news narrative? How did that make you feel? How did it compare with what you experienced?

**Reflections and Lessons Learned:**

- If there is one insight or lesson you wanted to share with others about your experience in the Navajo Nation what would it be?
- Are there any clinical insights or practices that you will bring home to UCSF following your experience working in the Navajo nation?
- (if not answered in above questions) What message or lessons do you have for society based on your experiences working in the Navajo Nation

**Academic Community:**

- If other academic medical centers are going to this type of work, deployment, partnership – what advice would you give them?
- Trust is the foundation to authentic partnerships – how do academic medical centers build trust with communities such as those in the Navajo Nation?
- How do academic medical centers (such as UCSF) prepare its workforce for efforts such as working and partnering with the Navajo Nation?
- How does UCSF build sustainable relationships and partnerships with the Navajo Nation?

**Wrap Up:**

- We have asked you a lot of questions, do you have any for us?

**Interview Guide: Volunteers who were administrative only**

**Introductions**

Overview of the project and aims

**The Basics**

- How were you first approached about sending providers to the Navajo Nation? Why did you say yes?

**Narrative & Partnership**

- The HEAL initiative talks about walking in solidarity with communities. Can you tell us about how that statement informed your planning for Navajo Nation, perhaps different than the planning that went into sending people to New York?
- What factors or experiences helped you facilitate a successful partnership with providers or administrators on the ground?
- What went well and what could have gone better in this process?
- What about the media/news narrative? How was that planned and designed?

**Academic Community:**

- If other academic medical centers are going to this type of work, deployment, partnership – what advice would you give them?
- Trust is the foundation to authentic partnerships – how do academic medical centers build trust with vulnerable communities such as those in the Navaho Nation?
- How do academic medical centers (such as UCSF) prepare its workforce for efforts such as working and partnering with the Navajo Nation?
- (if not answered in previous responses) How does UCSF build sustainable relationships and partnerships with the Navajo Nation?
- There are lots of financial concerns post-pandemic for all medical institutions. Given these pressures, how can medical institutions still conduct deployments to the Navajo Nation and why is this still important.

**Wrap Up:**

- Is there anything we’ve not covered that you would like to tell us about this initiative?

We have asked you a lot of questions, do you have any for us?
