# Supplementary material for: Lessons learned from academic medical centers’ response to the COVID-19 pandemic in partnership with the Navajo Nation
Source: PLoS One. 2022 Apr 5;17(4):e0265945. doi: 10.1371/journal.pone.0265945 (PMC8982841; doi:10.1371/journal.pone.0265945)
Supplement: S2 File — (DOCX) [file pone.0265945.s002.docx]

Supporting File 2: Supporting Quotes

1. **Mission and values: Alignment between the mission and values of the AMC with the crisis response effort is essential at every level of the organization. This alignment has great benefits at both the organizational and individual level.**

Civic duty: Many AMCs are thought to have a civic duty to health equity, to create and sustain partnerships with diverse and vulnerable communities. Partnering with the Navajo Nation is an example of how this could be achieved.

I think most people see AMCs as highly technical, complicated places that you want to have around if you have a very complicated disease that requires huge expertise and maximal technical experience. They don't really appreciate… how important they are in a public health crisis. To be a hub-and-spokes-type relationship with the academic medical centers being a hub but having spokes out into the community and particularly to the rural community where hospitals are.

We have an obligation outside of here to the state of California, to the nation, in ways that we should honor and always be ready for. We do have an obligation to be able to help and support wherever is needed…So, understand what your purpose is when you work in an AMC.

Come with an open mind but also come to engage in these partnerships, not to advertise them and not because the historical moment demands them but because it’s part of the culture and values of the institution or the system.

Real credit to our leaders who said, "We're going to make it possible because this is our mission, and we have to give truth to what we say our values are as an institution."

UCSF has a mission for addressing health inequity, and what an amazing opportunity to put resources and put our actions behind something that we have always espoused to be our mission.

Community engagement is a core value: AMCs must be willing to commit to community engagement as a core value, for the values sake, and not for positive publicity.

So that UCSF is perceived by the community as being responsive to their needs and not simply – I mean, I'm sure they take some pride if they hear that we won a Nobel Prize. There is another opportunity and I hope by working through this anchor institution concept we can change how the university is viewed by the community.

The academics who want to get involved, get involved. Not just during when it's cool to do it… but, you know, chairs hopefully will get engaged when there's not just the potential for a — a good tweet or a — a spot on the local news.

AMC leadership must be committed to activities that fulfil the mission and values of the organization: AMC leadership must be actively involved in and show commitment to, the values espoused by their organization related to community engagement and health equity.

We did a great job preparing [for the pandemic], but we then were able to kind of shift the focus to be external, like "What else is going on out there? Who else might need help?" That is rare. I think that's more of a mentality that's a lot harder to teach. I think it's something you can encourage in other institutions, but it is built in, I think, to the people, to the leadership. I think that it speaks volumes that, instead of hoarding and protecting what is ours, we were able to give freely and encourage service elsewhere.”

I just think it's really truly amazing and really speaks to UCSF's commitment to global health that they engaged in this. And engaged in this fully and completely and wholly. They didn't just half do it. UCSF leadership were on the weekly check-in calls. They went to the airport to send everybody off and wish them well. I really truly think that, certainly for me and my career, this is always something that I'll look back and feel very humble to have been a part of, and to have been able to help support this initiative. For UCSF to actually do this and do this in the caring, thoughtful, and supportive way that they did, and send one wave of volunteers, and then extend them, and then send another wave of volunteers, I think it was pretty incredible.”

Employees want to live out these values: Employee’s individual values act to reinforce the partnership and the organizational values, while also strengthening internal bonds between employees and their institution.

Going in to COVID I wasn't sure that UCSF was aligned with my values. I wasn't sure this was my forever academic home. And after seeing UCSF's response to Navajo Nation, or after being a provider there myself and feeling so supported by UCSF in undertaking this work, this has fundamentally changed how I feel about UCSF as an organization and as a community that I am so proud to be a part of. For me, and I hope for our UCSF leaders, that is worth the investment. That ability to rekindle the sense of community at UCSF and that ability to demonstrate that we can put our values that we have on paper into action.

I think it's really important to think about where AMCs are post-pandemic. There are a lot of financial constraints now on AMCs that might hinder them from embarking on this. And so, I think one question is, "For a now financially-strapped academic medical center why do this? "Why is this still a good idea? Why is this still important to do as an academic medical center?" I think when you're looking at what makes up an AMC and what makes AMCs special? When we ask people that question over and over again they say, "It's the people. It's the people who work here who may take a salary cut to work here who align with our values and our mission. That is what makes UCSF special." The response that I got from the providers and from the community at large about how this made them feel about UCSF, you can't put a price tag on that.

1. **Solidarity, trust and humility: Participants felt that building partnerships based on solidarity and trust** **was a cornerstone to the crisis response effort. This partnership was possible due to a pre-existing relationship that HEAL had with the Navajo Nation, which built trust over time. However, participants also advocated for a sustainable commitment to the Navajo people beyond the COVID-19 crisis. For success, partnerships require humility of the AMC and erasing the ‘savior’ narrative.**

Pre-existing trust from Navajo Nation partners: The rapid pandemic response was possible due to the pre-existing relationship and the commitment to the Navajo Nation. This commitment was trust-building.

I think what probably helped us in this scenario was the fact that HEAL was already established in the Navajo Nation. They had been there for years already, so there was a sense of understanding of what the purpose and the mission was of HEAL, that it was not just like a quick fix. We're here to plug your holes, and we're here to leave. It's about a matter of how we can continue this relationship over an extended period of time and addressing what is the best way for us to help rather than saying, "Okay, we're here to work for three months and then we're leaving.”

For organizations who want to leverage their own power to be there in solidarity with partners in resource-denied settings—the lesson is, build those deep partnerships that are real now. So that when your assistance is needed down the road, you'll been in a position to not try to build five years of trust in a day. But they'll be in a position to respond in the way that the partner would respect and would feel that it's value in that moment.

Trust often comes with relationships over time. Trust comes from previous agreements where responsibilities on both sides have been fulfilled. And so, if you don't have those relationships and you don't have that history of collaboration where both sides have met the expectations of the other it is hard to form that trust immediately. So, I think you would need to figure out how do replicate that in a really short timeframe.

Creating workforce sustainability within engagement plans: Participants advocated that the commitment and partnership would be sustained. This could be achieved by longitudinal workforce planning that went beyond relying on volunteerism.

I think the key is continuing the partnership. It needs to be support that is provided not just during this, you know, worldwide crisis, but somehow ongoing. I think everyone wants to help right now and, you know, the house is burning down, everyone's going to be there trying to help, but there are issues that are happening even though the world isn't paying attention, and I think you have to be there for those moments as well to show that you truly are invested and truly are walking in solidarity.”

Couldn't we get some sort of commitment as to how many residents, or if that's too tricky, how many faculty would be willing to be spread out over the next calendar year so that we're not just there to fly in at the peak of a crisis but can be for the ramp-up and ramp-down period, and really establish a long-term relationship that's more likely to -- you know, will just magnify the impact that they would have in the long run?

So rather than looking to fit in holiday time or vacation time but to really honor it and create a job that is funded adequately and that is providing benefits so that it’s not a tremendous ask for people to go...and it’s part of your FTE. I would say, sign me up. Where do I sign? I would happily do that. So creating it as part of my job, my career, making it part of my advancement -- somehow connecting it to the other non-clinical activities and academic research that I’m interested in doing, especially for people who want to work in social medicine or want in some way to be at a medical center but to really do social medicine…and I think making it a long-term relationship with a stable group of people will also go a long way to proving to the Nation -- the medical centers but also leadership there -- that it is sincere and it is not going to come and go. And it will be there year-in, year-out. The faces may change, but there will be, always, a presence [from the AMC].

Humility is key: To achieve solidarity, participants had to first identify and acknowledge their own humility. In order for the partnership to be honored, power hierarchies needed to be flattened, which required humility on the part of all clinicians and leaders from the AMC.

I think a lot it is just focused on listening to your partners. To the folks you want to partner with. I think so often, and so much more common than our model at HEAL, is what really ends up being like a replication of a colonial dynamic of very highly powered institutions—academia—with a lot of expertise in some frame, going in and saying, “Hey we have these people; they're highly skilled. We're going to send them to you, and we want them to do X, Y, and Z…” And, you know, that looks like the highly-powered institution calling so many of the shots. Which is really, in essence, a repetition of how global health has been done wrong, in many areas over history. And so, converse to that, it looks like approaching a partner with the community that you want to work and serve—and really listening and asking, kind of, “What do you need? And, you know, we have all these things to offer. What would be most helpful? And in what way? And what are we building towards in the future?” Not just in the short-term. And being prepared when the answer comes back a little bit different than what you'd kind of drawn out being prepared to respond to that.

It's like approaching in a humble way. Learning about the community that you're going to be partnering with. Listening and centering people's voices who are from that community, even if it doesn't always align with what we think is the most important thing or what we necessarily want. Then, understanding that it is important to approach this in a relational way.

Remind people to be humble. Humility, I think, is something that—because we're experts, we sometimes forget to how to be humble. And we barge in, then we replicate the cycle that's been done in the past. But I think if you are humble and open to what they have to offer and see how that can be incorporated into what we have. It's bridging the two, together. Because there's always a commonality, and there's always a way to bridge things and come to a common point. I think part of what made it work for us was we were humble. We were, you know, 'You want us to do five nights in a row? Sure, bring it on.'

“Savior narratives” need to be erased: AMCs need to be present in solidarity, not with a savior narrative (a common trope wherein a white protagonist is portrayed as a messianic figure who rescues non-whites from unfortunate circumstances).^20^

I think for me, it was just really aligning with the mission and really showing support for how resilient the community has been in managing it up until the point. So that was really our goal and our mission, to make sure that we're constantly reinforcing and supporting our staff and just being there to maybe fill in the gaps that have been left by sick healthcare providers or staffing issues.

I think my experience with the volunteers, really, most of whom did not have much experience in Navajo Nation, that I think having that framework coming in and we made it pretty clear, I think, from the beginning what our role was and what it wasn't, and really trying to take that savior lens off of the situation, and [they're like], "Nope, actually, we're just here to be an extra pair of hands" was really valuable.

It was really helpful to have a lot of background work from HEAL, and to not have the whole "white savior" narrative going on, and not have that whole dynamic of: "Well, we're going to come in. We are from this great medical center with all our great knowledge, and we're going to impart all this knowledge on you." It was more like: "We have more extra hands. We're here to help you right now for a short period. We'll do what we can. We'll do what you want us to do." I just made a point when I first got there -- and I kept trying to remind myself to do it, too: When people would thank me for coming, I would say: "Thank you for letting us be here." And I really meant it.

1. **Coordination: Many respondents spoke about the role of AMCs in making administrative, workforce and logistical arrangements related to travel, lodging, supplies and credentialing of clinicians. This included coordinating human resources issues and support for AMCs to support clinician time away from their primary site of work.**

Coordination of the crisis response: High quality administrative and logistical support is essential for rapid deployment of clinicians.

The challenge will never be interest. It will never be a lack of people who are willing and really highly interested in doing this work. The logistics is always key. Originally, I was going to be able to go for a week, and that seemed like it was going to be enough, but then they're like, "No, no. You have to come for at least two weeks or else it's not worth it to go through all the paperwork." It wasn't clear if my department was going to cover my shifts…This was the nature of doing something in a hurry but for the future, I would say that, to the extent [that it's] possible to have a streamlined approach or a person or team, if there was something in place that was like a framework, it would be nice to have that.

The reentry process was probably one of the biggest things that we had to really work on with regards to workflows. And then, working with labor and employee relations to be able to help us and allow for people to be able to take that 14 days as just reentry support, even if they didn't have a high-risk exposure that meant that they needed to quarantine. I think that was probably the biggest policy piece. But the Chancellor actually had to create a temporary assignment protocol that gave us some guidance around payment and nonpayment and all of that stuff.

I think one of the biggest things is getting the logistics right, and really thinking through all of those HR workflows. Because small things become big things when you're in another community that speaks another language, and you don't have good internet, and you don't have good phone service. Those small things can become big things that can affect your ability to be able to focus on your job and focus on the reason why you're there. Getting those logistical workflows on point and thinking through all of those different scenarios is really, really a key part of the deployment.

We realized that we needed to know things as we solicited volunteers and that we then needed to talk to Occupational Health. We had to have a logistics team for housing and transportation. We needed to talk to our PPE suppliers. We needed to think about professional services agreements and make sure that Worker's Comp and professional liability would be covered.

About their orientations. About the EMR. Whatever questions we could think about we put it in an indexed FAQ. And then, close to the deployment date we had a call, and we made sure that Occupational Health, Spiritual Health, Academic Affairs, Credentialing, all the people who came together to make this happen were on that call to be able to answer any questions the volunteers had.

Flexibility: The workforce needs to be willing to work wherever, and however, they are needed by the community health system.

On my unit, they had been experiencing the COVID surge for a month and a half already. So, me coming in and not consuming a lot of their resources by saying, "I'm going to need a week of orientation; you're going to have to tell me how to do everything," The fact that I could actively contribute right away dispersed some of the burden of the heavy patient caseload or the high census. I think they really appreciated that. It was like, "Great, now that you're here, we don't have to take as many patients among us. We can distribute it a little bit more manageably”.

For our volunteers, we tried to support them by guiding them to see which department needed skills most. Folks … were passed into other departments—their high degree of training and expertise and their flexibility and resilience were able to be slotted into different departments than the ones that they are in at UCSF. And so, A) go wherever you're needed, and then B), once you're there, do whatever is needed. It isn't time to question protocols, it isn't time to mention the way this would be done at UCSF, it's the way to actually be alongside and support.

We were, you know, 'You want us to do five nights in a row? Sure, bring it on.'

It's arriving, and finding out what the local priority is, and it may have nothing to do with your area of expertise and being willing to go and do that. And so, that was — that's a big part of the screening process, trying to …get a sense of how well somebody might adapt if you're a highly trained intensivist and anesthesiologist, and you are going there.

Selectivity about who and what is sent: AMCs need to make sure they are sending the appropriate personnel and equipment needed by the partner, as opposed to sending items the AMC might have available. Balance is important.

There’s the impulse to send a bunch of stuff but, it’s hard to manage all that stuff that needs to be inventoried and tracked. And then the donor wants to know what it was used for. And then the huge burden to ask an already fairly resource-poor area to start to handle 3,000 individual beef jerky. I was the last one there… I had to empty the storage room in the hotel where everyone had brought stuff and left it in this room. I had to figure out where all that stuff went and who would take it over. Should I throw away, take them with me, or should I leave them?

I could tell from the people who told me, "Well, I want this certain shift and I want this certain [thing]." "No, you can't go then because you think it's going to be like here, and that's not going to be the case." For the Navajo Nation, I would just reject people [during screening] because I just said, "You're not ready for this." Because you have to do what is needed at all times at the end of the day.

What's most useful is to help organize the PPE closet and make sure everything is clean and easily accessible. And to do that, you know, 10 hours a day. And for some people that's a non-starter. And for other people, it's not. That's not exactly how that question is phrased to potential volunteers. But that — that is an important part of trying to suss it out. You know, what — what exactly are the expectations. How do they think they might be the most useful?

I think that it’s worthwhile vetting people carefully who go there so that you’re really making sure they’re aligned with what you want to do and that you are really protecting that relationship so that the work can be the best it can be.

Media preparation: Clinicians struggled with how to respond to the media. AMCs need to help coordinate and prepare clinicians for potential media enquiries so that the mission and narrative is not usurped by the media.

Watching the news clip, they literally took what I said out of context in order to fit the narrative of how they wanted to portray what was happening in the Navajo Nation. I thought that was really concerning because it wasn't portraying an accurate picture of the situation; it was more in terms of, "Look at how these people from San Francisco are coming in to save the day." They would have clips of President Nez just crying and saying, "We're in trouble, we need help, we can't do this on our own." Taking those interviews and taking those clips out of context to fit this more widely accepted narrative that America's in great shape, and we're here to save the day really devalues the resilience and the integrity of the Navajo Nation and what they've experienced during this pandemic.

I think there is danger in the narrative, it just perpetuates the saviorism, must save this group of people. No, we don't need to save them, we need to stop oppressing them. It's just not talking about like the structural oppression and issues, like why do 30 percent of the people not have electricity and running water?

Most of it was pretty cringey. When we got to the airport in Albuquerque there was a reporter who interviewed a couple people. They created this story: "Oh, these great, awesome medical people are here from San Francisco." It was the total "white savior" thing. The very first patient that I had, I told her I was from California, and she [said]: "Oh, are you with that group that was on the TV.

1. **Workforce preparation and support**: **Pre-departure preparation of the workforce was critical. This included ensuring clinicians are educated about the historical context of broken promises and trust on the Navajo Nation, the culture of the Navajo people, the healthcare delivery system, and the persistent health inequalities that exist. Further workforce preparation was needed around the concept of health equity, solidarity and how to reject the savior narrative. Workforce support also required to clinicians were working on the Navajo Nation.**

Understanding the historical context of Native American communities: Clinicians expressed the need to have an understanding of the Navajo Nation and historical context of the Nation’s relationship with the United States.

Going to a new place. Really learning about the history of the people. And thinking about the whys behind a lot of the social differences. And the social determinants of health differences. That I definitely need more of.

When you're coming into an area, you do need to understand not only current but historical context, especially with things like COVID because COVID rates here didn't occur in a vacuum. There's a lot of systemic violence that has happened over a very long time that have created these conditions. Nothing occurs in a vacuum and we tend to be very ahistorical and acontextual. So, what is the historic and current context? Educating people what kind of system are we coming into because we know that our [healthcare] systems…are funded differently and work differently than an AMC type of system.

I think, in the same way that we're talking about being antiracist, we have to be anti-colonial, and we actually have to work to unbuild those structures.

There was one [background reading] about cultural humility that totally shifted my perspective on where we were going and why we were going there and how to be there with people. I think giving people a really explicit framework about just not going in with this very hierarchical -- expecting a very hierarchical, typical role.

Understanding healthcare in resource limited settings: Clinicians desired preparation on providing healthcare in resource limited settings.

One example is the electricity issue, so some patients have electricity that is there all the time, and other patients they live in homes where they have a generator that will give them electricity. And this is important because the oxygen compressors that we would send people home with you have to have electricity all the time, it can't be dependent on a generator because if the generator fails then you can't just like not be without oxygen if you're dependent on it. Understanding some of that living situation was really important. Also like how many people are in the home? were they all COVID exposed already? what would the risk be of sending someone home? Understanding those family dynamics is I think how I connected with patients.

Curriculum is not taught in isolation. It's taught with fellows, partners, from the partnering sites as well. Because it's not — it's not didactic. It's not transferring information. It's sharing information and trying to, you know, understand and assimilate with different perspectives that you wouldn't be able to teach without local expertise and context. So, somehow integrating that into the preparation.

Dangers of inadequate preparation: Some participants expressed that a lack of preparation could lead to harm through perpetuating past wrongs through misunderstandings of culture and context.

It would be nice to have been able to prepare people to go more than we were able to. Everything got operationalized in a very short period of time. In the course of a matter of days. We didn't really have adequate time to go through some of the ethical dilemma teaching that we often go through before practicing in resource-constrained settings with trainees and faculty. We didn't get a chance to talk through challenging scenarios that may come up that are relevant to resource-constrained practice settings.

I wish we had known more about this particular patient population in more detail prior to coming because we probably said a lot of things that maybe weren't appropriate. We probably approached patients in a way that we didn't realize was not the most sensitive just simply because we didn't know. I think having a very comprehensive understanding of what the patient population you are serving and what their needs are and how you as a clinician can observe and modify your clinical practice to be more sensitive to the needs of that community would be really, really helpful.

Without that deep structural analysis, there's such a deep risk in replicating the harm and, as outsiders going into a place like Navajo Nation, there are so many pitfalls around every corner. And if you don't go in with the proper contextual understanding of decisions that have been made, actively, by past institutions and the US Government, in this case, that have directly had implications on the health outcomes of this community. I think it's really important not to just do a cursory brief primer before traveling to a place, but to actually sit down and have discussions with colleagues who are educated in this area around what has happened, why, and what are some of the ways that we can help to address this that does replicate those dynamics that have caused harm in the first place. I felt like, if we had known that before coming here, maybe I would have approached these particular patients more sensitively or more holistically as soon as we got there.

Provision of emotional support: Providing emotional support for clinicians while working is essential but difficult in the midst of a pandemic.

So they go to work for 12 hours a day, there were weeks that I worked seven days a week. So, then you have this emotional like burden sitting on your chest while you're trying to do the work that you came to do.

[Support] looked like two or three times per week de-brief calls. Where we just held space for volunteers to process—to navigate hard encounters they'd had. We often times tried to bring in our own community members, who are already working at these sites. To be on these calls and to provide a little bit more of local context, to provide a different frame and a different lens for volunteers noticing a really challenging process or dynamic.

We learned quickly the value in the support that we were giving to our volunteers, and we really tried to hold ourselves honorable to that. I think that was probably one of the biggest learnings that we had quickly, was that it was really valuable for them to be able to just email me or text me, and for me to be able to take care of something for them. We also tried to set up regular weekly check-ins that, actually, senior leadership, the Chancellor and CEO and President also participated in. And to make sure that we were hearing about what was happening on the ground with them so that we'd be able to respond to anything that was coming up. We really, really saw the value of just having such close contact with the volunteers.

We took people from one community and put them in another community that was far from home. And we expected them to not have communal dinners. And socially distance and be perfect with their PPE. And we underestimated how much they would need a new community when they got there because this work was hard and often times, emotionally exhausting. We had some potential exposures as a result of that. Everything turned out okay but thinking about a pandemic and sending your providers to an unfamiliar space you have to make sure that they're going to be prepared for how isolating that may be. And figure out a way to give them a community and a safe space.
